# Prediabetes and Type 2 Diabetes are Independent Risk Factors for Computed Tomography-Estimated Nonalcoholic Fatty Pancreas Disease

**DOI:** 10.6061/clinics/2019/e1337

**Published:** 2019-10-23

**Authors:** Süleyman Ahbab, Ahmet Ünsal, Hayriye Esra Ataoğlu, Tuba Selçuk Can, Derya Kayaş, Yıldıray Savaş

**Affiliations:** I Internal Medicine Clinic, Haseki Training and Research Hospital, University of Health Sciences, Istanbul, Turkey; II Department of Radiology, Haseki Training and Research Hospital, University of Health Sciences, Istanbul, Turkey

**Keywords:** NonAlcoholic Fatty Pancreas Disease, Prediabetes, Type 2 Diabetes

## Abstract

**OBJECTIVES:**

Nonalcoholic fatty pancreas disease (NAFPD) is characterized by excessive fat deposition in the pancreas in the absence of alcohol consumption. In this study, we aimed to detect a possible relationship between adipose tissue accumulation, prediabetes and diabetes.

**METHODS:**

This cross-sectional and retrospective study included 110 patients. Three groups were classified as controls, patients with prediabetes and patients with type 2 diabetes. The abdominal computed tomography (CT) attenuation measurement results of the pancreas were evaluated independently by two experienced radiologists. CT measurements and biochemical parameters were compared between study groups. The relationship between continuous variables was assessed by using one-way ANOVA. To determine the changes in the dependent variable for the effects on study groups, the independent variable was adjusted using ANCOVA. A *p*-value less than 0.05 was considered statistically significant.

**RESULTS:**

The presence of prediabetes and type 2 diabetes was correlated with a decrease in the mean Hounsfield Unit (HU) value of the pancreas (*p*=0.002). Age was determined to be an independent risk factor and was correlated with NAFPD (*p*=0.0001). When compared to the controls (*p*=0.041), 71% of patients with prediabetes and 67% of patients with type 2 diabetes were observed to have an increased incidence of NAFPD. Decreased serum amylase was found to be correlated with the mean HU value of the pancreas (*p*=0.043).

**CONCLUSION:**

NAFPD was independently correlated with both prediabetes and type 2 diabetes adjusted for age (*p*=0.0001) in this study. Additionally, age was determined to be an independent risk factor and was correlated with NAFPD.

## INTRODUCTION

Obesity and diabetes are major health problems that lead to infiltration of visceral adipose tissue in addition to atherosclerotic cardiovascular diseases (1). Visceral fat deposition develops in organs such as the liver, pancreas and skeletal muscle as a result of ectopic adiposity (2). Nonalcoholic fatty pancreas disease (NAFPD) is closely associated with nonalcoholic fatty liver disease (NAFLD), diabetes, obesity and metabolic syndrome (3). NAFPD is excessive lipid deposition in the pancreas without alcohol consumption (4). Both low-grade inflammation and insulin resistance play an important role in the development of NAFPD. Sustained lipid accumulation in adipocytes causes elevated secretion of fat-derived proinflammatory molecules such as interleukin-6, tumor necrosis factor-α, and monocyte chemotactic protein-1 within pancreatic islets (5). Fatty infiltration related to inflammation can cause β-cell apoptosis, endocrine dysfunction and fibrosis of the pancreas. However, diabetes is known to have an important role in the progression of NAFPD (6-8), and there is limited data on the relationship between NAFPD and prediabetes, which is a metabolic parameter associated with insulin resistance. Prediabetes may promote NAFPD and pancreatic dysfunction. The aim of this study was to determine the relationships between NAFPD and both prediabetes and type 2 diabetes.

## METHODS

### Study participants

This retrospective study was conducted from January 2016 to January 2017 in Haseki Training and Research Hospital, University of Health Sciences in İstanbul. Haseki Training and Research Hospital’s local ethics committee approved the study design (Reference No: 03R/2018, Date: January 23^rd^, 2018). The database information was anonymized and approved by the ethics committee with no need for consent. Data for the study were derived from the electronic management system of the hospital. Patients selected for evaluation in this study had been admitted to our internal medicine outpatient clinic with complaints (such as abdominal pain, chronic dyspepsia, chronic constipation, etc.) and underwent an abdominal computed tomography (CT) scan during the differential diagnosis investigation as part of the clinician’s evaluation. Patients with acute abdominal syndrome, viral hepatitis, pancreatitis, hepatic cirrhosis, chronic renal disease, sepsis, chronic heart failure, malignancy, alcohol consumption, neurological and psychiatric disease were excluded from the study. In this study, a total of 110 patients were found to be eligible. Biochemical parameters [serum glycosylated hemoglobin (HbA1c), fasting blood glucose (FBG), creatinine, urea, hepatic and biliary enzymes, amylase, lipase, lipid profiles, albumin and c-reactive protein (CRP) levels] of the study patients were evaluated. Biochemical analysis was performed with an Abbott Architect Analyzer System (IL, USA). Three groups were included as nondiabetic controls (n=39), patients with prediabetes (n=43) and patients with type 2 diabetes (n=28). The study groups were classified according to the medical history of the participants, taking into account the hospital database records of patients with diabetes and prediabetes who were previously identified and receiving treatment. Prediabetes and type 2 diabetes mellitus were described according to the Standards of Medical Care in Diabetes 2018 criteria by the American Diabetes Association (9). Prediabetes was defined as patients with FBG between 5.6-6.9 mmol/L and/or HbA1c 39-47 mmol/mol. Diabetes mellitus was defined as patients who had FBG ≥7 mmol/L, 2 hours postprandial glucose ≥11.1 mmol/L and/or HbA1c ≥48 mmol/mol. The biochemical and CT results were compared among the study groups.

### Radiological evaluation

Abdominal CT scans were performed with a 64-detector Philips Brilliance CT device (Philips Medical Systems, Cleveland, Ohio). All the images were acquired according to a routine intravenous contrast-enhanced abdominal CT protocol (upper abdominal CT without axial contrast and whole abdominal CT taken in portal venous phase at 60 s). The shooting parameters were as follows: tube current 20 mAs; tube voltage, 120 kVp; pitch, 0.671; collimation, 64x0.625 mm; rotation time, 0.5s; cross-sectional thickness, 5 mm; and reconstruction range, 4 mm. Images were taken from pre-contrast axial sections using INFINITT PACS version 3.0.11.4 (INFINITT Healthcare Co. Ltd., Korea) by two expert radiologists with 5 years of experience. Radiologists were blinded to the patients’ clinical data. Attenuation values of the liver, spleen and pancreas were measured and expressed as Hounsfield Units (HUs). The attenuation measurements were performed using a 0.5 cm^2^ elliptical region of interest (ROI). Liver attenuation was determined as the average of the measurements made from the right, left and caudate lobes, and spleen attenuation was determined by taking the average of three measurements made from the lower, middle and upper parts of the spleen. During the assessment, care was taken not to measure formations that could affect the measurement, such as mass, cysts and calcification of the ROI. Attenuation measurements of the caput, corpus and cauda pancreas were separately recorded and evaluated independently by two experienced radiologists. The arithmetic mean of these three measurements was considered the mean attenuation HU value of the pancreas. Pancreatic vascular structures were not included in pancreatic attenuation measurements. CT-estimated NAFPD was defined as the difference in pancreas-spleen attenuation in negative HU value (10-13). The median was calculated according to the mean HU value of the pancreas of control patients. A cutoff value was determined (-4.00 HU, min: -17.00 and max: 21.00) to diagnose the CT-estimated incidence of NAFPD in patients with prediabetes and type 2 diabetes.

### Statistical analysis

Data were expressed as the mean ± standard deviation. SPSS 16.0 for Windows was used to perform statistical analysis. The distributions of variables were assessed by using the Kolmogorov-Smirnov Z test. T tests were used to analyze normally distributed variables, and the Mann-Whitney U test was used to analyze nonnormally distributed variables. The relationship between continuous variables was detected by using one-way ANOVA, and subgroup analysis was interpreted according to Bonferroni correction in parametric tests. The chi square test was used to evaluate categorical variables. Pearson and Spearman correlation analyses were performed to analyze the correlation between variables. When investigating changes in the dependent variable for the effects on study groups, the independent variable was adjusted using analysis of covariance (ANCOVA). A *p*-value less than 0.05 was considered statistically significant.

## RESULTS

A total of 991 patients who were admitted to the internal medicine clinic and underwent abdominal CT for any medical purpose as a further examination were investigated via the hospital’s medical database. Exclusion criteria were set to eliminate many diseases that may affect biochemical parameters and CT scan measurements of the pancreas and liver. Acute abdominal syndrome, viral hepatitis, pancreatitis, hepatic cirrhosis, chronic renal disease, sepsis, chronic heart failure, malignancy, alcohol consumption, and neuropsychiatric diseases were reasons for exclusion in this study. Following a detailed medical database investigation, 110 patients did not meet the exclusion criteria and were selected for the study. The characteristics and biochemical parameters of the study groups are shown in Table 1. The mean age of the control group was 55.28±14.19; patients with prediabetes, 63.88±14.97; and patients with type 2 diabetes, 65.18±11.02; the difference was statistically significant (*p*<0.005). The mean FBG and HbA1c levels were 5.05±0.36 mmol/L and 36.0±2.1 mmol/mol in controls, 5.91±0.80 mmol/L and 40.8±2.0 mmol/mol in patients with prediabetes and 7.80±3.67 mmol/L and 55.1±8.6 mmol/mol in patients with type 2 diabetes, respectively (*p*=0.0001 and 0.0001). The mean high-density lipoprotein (HDL) cholesterol was 1.30±0.30 mmol/L in controls, 1.19±0.29 mmol/L in patients with prediabetes and 1.10±0.36 mmol/L in patients with type 2 diabetes (*p*=0.035). Other biochemical parameters were not significantly different between groups. CT attenuation measurements of the liver, pancreas and spleen of the study patients are shown in Table 2. A decrease in pancreatic attenuation and a negative HU value between the pancreas and spleen were diagnostic for NAFPD. Caput, corpus, cauda and mean HU values of the pancreas were significantly decreased in patients with prediabetes and type 2 diabetes. Mean HU attenuation differences between the parts of the pancreas and spleen were statistically significant between groups. A significant increase in pancreatic fat accumulation was found in both patients with prediabetes and those with type 2 diabetes compared to controls (*p*=0.041). Caput, corpus, cauda and mean HU values of the pancreas were observed to correlate with age, FBG and HbA1c, as shown in Table 3. Serum CRP levels were significantly elevated in patients with prediabetes and those with type 2 diabetes compared with controls (*p*=0.004). On the other hand, CRP levels were also categorized according to our laboratory’s cutoff value (47.62 nmol/L) because of the wide range of distribution. Serum amylase levels correlated with corpus, cauda and mean HU value of the pancreas. Mean HDL cholesterol and triglyceride levels were found to correlate with only the attenuation HU of liver. ANCOVA was performed to reveal the effect of type 2 diabetes and prediabetes on the mean HU value of the pancreas adjusted for age. The presence of type 2 diabetes and prediabetes correlated with a decrease in the mean HU value of the pancreas (*p*=0.002), as shown in Table 4. A contrast hypothesis (K Matrix) was applied to compare the relationship of the mean HU values of the pancreas between patients with prediabetes versus controls (Level 2 *vs*. Level 1) and patients with type 2 diabetes versus controls (Level 3 *vs*. Level 1). Contrast results were statistically significant (Table 5); *p*=0.015, 95% CI:-1.117 and -10.483 for Level 2 *vs*. Level 1, and *p*=0.001, 95% CI:-3.987 and -14.431 for Level 3 *vs*. Level 1.

## DISCUSSION

Prediabetes and diabetes are associated with visceral adipose tissue accumulation, especially in the pancreas and liver, and has important clinical consequences in addition to atherosclerosis. NAFPD varies from simple fat storage and inflammation to the development of pancreatic fibrosis. NAFPD is one of the manifestations of type 2 diabetes and metabolic syndrome (14). We found that NAFPD independently correlated with both prediabetes and type 2 diabetes adjusted for age and that aging is an important risk factor for fatty pancreas. Moreover, we observed that NAFPD is widespread in all parts of the pancreas, not only in specific regions. ANCOVA is a useful statistical technique for eliminating the effect of a different numerical variable (such as age) during the comparison of the means of a variable in two or more groups. As a result of ANCOVA, prediabetes and diabetes were found to be independently related to the development of NAFPD. Contrast results revealed that the presence of prediabetes and diabetes were consistent with the increase in pancreatic fat content. Furthermore, prediabetes and diabetes were found to have an independent role in the progression of visceral fat accumulation and NAFPD (14). Ou et al. also indicated that NAFPD was associated with insulin resistance, obesity, prediabetes, metabolic syndrome and diabetes (15-19). Steatosis of the pancreas with triglyceride accumulation can lead to a decline in β-cell mass and function, and potentially lead to the development of diabetes (20). Moreover, NAFPD may develop with aging as well as with diabetes mellitus, and age was found to be an independent risk factor and was correlated with NAFPD (*p*=0.0001). However, 46% of control patients were diagnosed with NAFPD according to our study, although the incidence of NAFPD was significantly higher in patients with diabetes and prediabetes than in controls. Weng et al. reported that the occurrence of NAFPD increases independently with age, obesity and diabetes (21). Advanced age is associated with pancreatic fat deposition and plays an important role in pancreatic atrophy and fibrosis (22,23). On the other hand, the detection of NAFPD in younger control patients may indicate that NAFPD begins to develop at an earlier age.

In this study, NAFPD incidence was observed in 71% of patients with prediabetes and 67% of patients with type 2 diabetes, which was significantly higher than in the controls. Furthermore, NAFPD was found to correlate with FBG and HbA1c levels in our study. Consistent with our findings, Wu et al. suggested that metabolic parameters such as abdominal obesity, FBG and HbA1c were strongly associated with fatty pancreas (24). CRP is an important marker of inflammation. In our study, the rates of high CRP levels were 10/39 (25.6%) in controls, 15/43 (34.3%) in patients with prediabetes and 18/28 (64.3%) in patients with type 2 diabetes. Categorized CRP levels were elevated in patients with type 2 diabetes and prediabetes compared with controls. Moreover, CRP levels were higher in patients with prediabetes and type 2 diabetes than in controls in our study, suggesting a relationship between inflammation and NAFPD.

To accurately identify NAFPD, CT is an approved method for evaluating pancreatic fat accumulation with or without contrast and is easily applicable. The density of pancreatic steatosis was similar to the density of adipose tissue on CT scan using HU (24). Although there was a decrease in the mean HU value of the liver, it was not found to be significant. van Geenen et al. reported that insulin resistance and obesity play an important role in steatosis of the liver and pancreas and adipocyte infiltration. NAFPD and NAFLD are both a result of insulin resistance (25). NAFPD is a predictor of NAFLD and is related to hepatic steatosis rather than total body fat. Central obesity is closely associated with fatty liver and pancreas in patients with type 2 diabetes and prediabetes (26). Lee et al. suggested a possible relationship between NAFPD and NAFLD (27). Notably, NAFLD is associated with metabolic syndrome and clinical consequences (28). Moreover, fatty liver was correlated with increased FBG, triglyceride and decreased HDL in this study. The total cholesterol level was higher in controls than in patients with prediabetes and type 2 diabetes, although the difference was not statistically significant. This finding may be a result of the patients with type 2 diabetes and prediabetes paying attention to their diet. Consistent with this finding, LDL cholesterol levels were decreased in patients with diabetes compared with participants in the other groups, although the difference was not statistically significant. The prevalence of impaired glucose tolerance and diabetes is elevated in NAFLD patients (29,30).

The mean HU values of the pancreas were significantly correlated with a decrease in serum amylase levels. In other words, fat accumulation in the pancreas was correlated with low serum amylase levels. Amylase is an indicator of the exocrine function of the pancreas. Increased triglyceride content of pancreatic tissue enhances pancreatic expression of a fibrogenic marker (TGF-β) and collagen production (31). NAFPD is associated with a decrease in both endocrine and exocrine functions of the pancreas as a result of inflammation and fibrosis (32,33). Therefore, the correlation between a decreased amylase level and mean pancreas HU value may be considered a result of the onset of an insufficiency in pancreatic exocrine functions in our study.

Notably, this study has some limitations. First, this cross-sectional retrospective study was based only on CT scan measurements of the pancreas and liver. Medical records and CT results were examined electronically on the computer. Radiological methods other than CT may be used to analyze NAFPD as an external validation. However, anthropometric measurements of all patients could not be obtained, and serum insulin levels were not analyzed for all study patients. Therefore, insulin resistance values were not provided from the medical records. Insulin resistance and anthropometric measurements are important parameters for identifying the presence of metabolic syndrome. Although the results were age adjusted, the relationship between NAFPD and metabolic parameters could not be evaluated because of the study design. The results of this study will provide ideas for new research, and further studies are needed with a larger number of patients.

## CONCLUSION

In conclusion, NAFPD was independently correlated with both prediabetes and type 2 diabetes adjusted for age (*p*=0.0001) in this study. Additionally, age was an independent risk factor and correlated with NAFPD. Further studies are needed to investigate the relationship between anthropometric measurements and NAFPD.

## AUTHOR CONTRIBUTIONS

Ahbab S designed the study and contributed to formal analysis and writing-original draft. Ünsal A contributed to the investigation, methodology and writing-original draft. Ataoğlu HE contributed to data curation, formal analysis and investigation. Kayaş D contributed to data curation. Can TS and Savaş Y performed radiological evaluation, investigation and visualization.

## Figures and Tables

**Table 1:** General characteristics and biochemical parameters of the study groups.

Parameters (laboratory ranges)	Controls (n: 39)	Patients with prediabetes (n: 43)	Patients with type 2 diabetes (n: 28)	*p-*value
Age	55.28±14.19	63.88±14.97	65.18±11.02	***0.005***
FBG (3.9-6.1 mmol/L)	5.05±0.36	5.91±0.80	7.80±3.67	***0.0001***
HbA1c (20-48 mmol/mol)	36.0±2.1	40.8±2.0	55.1±8.6	***0.0001***
ALT (0.17-0.68 µkat/L)	0.39±0.23	0.60±1.16	0.37±0.17	0.32
AST (0.17-0.58 µkat/L)	0.39±0.10	0.55±0.84	0.35±0.11	0.23
Amylase (0.46-1.67 µkat/L)	1.44±0.84	1.28±0.40	1.32±0.80	0.56
Lipase (0.08-1.12 µkat/L)	0.57±0.70	0.41±0.29	0.74±0.99	0.18
GGT (0.03-0.62 µkat/L)	0.45±0.24	1.12±3.01	0.55±0.35	0.24
ALP (0.5-2.0 µkat/L)	1.34±0.33	1.50±0.78	1.61±0.67	0.20
Total cholesterol (<5.18 mmol/L)	5.46±1.19	5.10±1.23	4.79±1.08	0.076
HDL cholesterol (>1.03 mmol/L)	1.30±0.30	1.19±0.29	1.10±0.36	***0.035***
LDL cholesterol (<4.14 mmol/L)	3.44±0.95	3.10±1.05	2.91±0.97	0.08
Triglyceride (<1.69 mmol/L)	1.56±0.71	1.85±0.99	1.71±0.80	0.32
LDH (1.7-4.12 µkat/L)	3.10±0.49	3.14±0.57	3.04±0.82	0.79
Albumin (35-50 g/L)	43.30±3.60	42.20±3.20	41.00±4.70	0.06
CRP (<47.62 nmol/L)	55.14±99.53	110.76±197.43	237.72±337.53	***0.004***
Hypertensive patients (n, %)	10/39, 25%	12/43, 27%	9/28, 32%	0.56

(FBG: fasting blood glucose, HbA1c: glycosylated hemoglobin, ALT: alanine aminotransferase, AST: aspartate aminotransferase, GGT: gamma glutamyl transferase, ALP: alkaline phosphatase, HDL: high-density lipoprotein, LDL: low-density lipoprotein, LDH: lactate dehydrogenase, CRP: c-reactive protein)

(statistically significant *p*-values were expressed in bold and italic)

**Table 2:** A comparison of CT attenuation measurements of the liver, parts of the pancreas and spleen between the patient groups.

	Controls (n: 39)	Patients with prediabetes (n: 43)	Patients with type 2 diabetes (n: 28)	*p*-value
Liver (HU)	55.37±8.93	53.15±7.63	50.21±10.75	0.072
Caput Pancreas (HU)	44.03±5.96	35.72±12.82	31.96±14.50	***0.0001***
Corpus Pancreas (HU)	43.59±7.17	35.00±12.29	30.61±14.61	***0.0001***
Cauda Pancreas (HU)	41.69±7.68	34.51±12.28	31.50±13.28	***0.001***
Mean HU of Pancreas	43.09±6.19	35.05±11.85	31.34±13.72	***0.0001***
Spleen (HU)	48.69±4.50	47.05±5.57	45.39±6.22	***0.05***
Caput P-S value (HU)	-4.66±7.19	-11.32±12.01	-13.42±16.94	***0.008***
Corpus P-S value (HU)	-5.10±8.21	-12.04±12.47	-14.78±16.78	***0.005***
Cauda P-S value (HU)	-7.00±8.03	-12.53±12.15	-13.89±15.05	***0.035***
Mean HU of P-S value	-5.60±7.14	-11.98±11.58	-14.04±15.91	***0.008***
NAFPD (n, %)	18/39, 46%	31/43, 72%	19/28, 67%	***0.041***

(HU: Hounsfield unit, P-S value: difference between HU values of part of pancreas and spleen, NAFPD: nonalcoholic fatty pancreas disease, n: number of patients)

(statistically significant *p*-values were expressed in bold and italic)

**Table 3:** Correlations between age, biochemical parameters and CT-estimated HU values of liver and pancreas.

		Age	FBG	HbA1c	Amylase	Triglyceride	HDL
Liver (HU)	***r***	0.039	-0.221	-0.28	-0.10	-0.278	0.201
	***p***	0.682	***0.02***	***0.004***	0.297	***0.004***	***0.037***
Caput Pancreas (HU)	***r***	-0.363	-0.295	-0.464	0.167	-0.02	0.121
	***p***	***0.0001***	***0.002***	***0.0001***	0.082	0.839	0.212
Corpus Pancreas (HU)	***r***	-0.390	-0.304	-0.498	0.189	-0.049	0.125
	***p***	***0.0001***	***0.001***	***0.0001***	***0.048***	0.612	0.198
Cauda Pancreas (HU)	***r***	-0.420	-0.274	-0.464	0.20	-0.035	0.068
	***p***	***0.0001***	***0.004***	***0.0001***	***0.036***	0.72	0.486
Mean HU of Pancreas	***r***	-0.408	-0.304	-0,496	0.194	-0.036	0.11
	***p***	***0.0001***	***0.001***	***0.0001***	***0.043***	0.711	0.259
Caput P-S value (HU)	***r***	-0.341	-0.156	-0.347	0.125	0.052	0.041
	***p***	***0.0001***	0.103	***0.0001***	0.195	0.593	0.672
Corpus P-S value (HU)	***r***	-0.361	-0.164	-0.375	0.144	0.021	0.045
	***p***	***0.0001***	0.087	***0.0001***	0.133	0.827	0.642
Cauda P-S value (HU)	***r***	-0.398	-0.131	-0.345	0.156	0.04	-0.015
	***p***	***0.0001***	0.174	***0.0001***	0.103	0.679	0.881
Mean P-S value (HU)	***r***	-0.382	-0.157	-0.37	0.148	0.039	0.026
	***p***	***0.0001***	0.102	***0.0001***	0.124	0.686	0.793

(HU: Hounsfield unit, P-S value: difference between HU values of part of pancreas and spleen, FBG: fasting blood glucose, HbaA1c: glycosylated hemoglobin, HDL: high-density lipoprotein)

(statistically significant *p*-values were expressed in bold and italic)

**Table 4:** ANCOVA between-subjects effects. The dependent variable is the mean HU value of the pancreas adjusted for age.

Source	Type III Sum of Squares	df	Mean Square	F	*p*-value
Corrected Model	3838.799^a^	3	1279.600	12.204	0.0001
Intercept	13996.312	1	13996.312	133.492	0.0001
Age	1335.012	1	1335.012	12.733	***0.001***
Study Groups	1353.861	2	676.930	6.456	***0.002***
Error	11113.854	106	104.848		
Total	165224.621	110			
Corrected Total	14952.653	109			

a R Squared = 0.257 (Adjusted R Squared = 0.236)

(HU: Hounsfield Unit, statistically significant *p-*values were expressed in bold and italic)

**Table 5:** Contrast hypothesis results between study groups as patients with prediabetes *vs*. controls (Level 2 *vs*. Level 1) and patients with type 2 diabetes *vs*. controls (Level 3 *vs*. Level 1).

Contrast Results (K Matrix)			
Study Groups Simple Contrast^a^			Dependent Variable Mean HU of Pancreas
Level 2 *vs*. Level 1	Contrast Estimate		-5.830
	Hypothesized Value		0
	Difference (Estimate - Hypothesized)		-5.830
	Standard Error		2.347
	***p***-value		***0.015***
	95% Confidence Interval for Difference	Lower Bound	-10.483
		Upper Bound	-1.177
Level 3 *vs*. Level 1	Contrast Estimate		-9.209
	Hypothesized Value		0
	Difference (Estimate - Hypothesized)		-9.209
	Standard Error		2.634
	*p*-value		***0.001***
	95% Confidence Interval for Difference	Lower Bound	-14.431
		Upper Bound	-3.987

a Reference category = 1

(HU: Hounsfield Unit, statistically significant *p*-values were expressed in bold and italic)
